# Ohio State Dental Society

**Published:** 1893-02

**Authors:** 


### OHIO STATE DENTAL SOCIETY.

At the Ohio State Dental Society, held at Columbus, December 6-9, '92, the officers elected were as follows:

President, G. H. Wilson, Cleveland; 1st Vice President, Chas. Welch, Wilmington; 2nd Vice President, W. H. Todd, Columbus; Secretary, L. P. Bethel, Kent; Assistant Secretary, Henry Barnes, Cleveland.

